# Presence and Plant Uptake of Heavy Metals in Tidal Marsh Wetland Soils

**DOI:** 10.3389/fpubh.2022.821892

**Published:** 2022-02-21

**Authors:** Lathadevi K. Chintapenta, Katharine I. Ommanney, Gulnihal Ozbay

**Affiliations:** ^1^Department of Biology, University of Wisconsin River Falls, River Falls, WI, United States; ^2^Department of Agriculture and Natural Resources, Sciences, Delaware State University, Dover, DE, United States

**Keywords:** heavy metals, arsenic, cadmium, lead, marsh grass, *Spartina alterniflora*, *Phragmites australis*

## Abstract

Marsh grasses have been used as efficient tools for phytoremediation and are known to play key roles in maintaining ecosystem functions by reducing the contamination of coastlines. This study was initiated to understand how human activities in wetlands can impact ion-heavy metal concentrations in relation to native and invasive marsh grasses. The study site, Blackbird Creek (BBC) is a tidal wetland that experiences agricultural, fishing, recreational, residential and other anthropogenic activities throughout the year. Heavy metals cadmium, arsenic, and lead in the soils and marsh grasses were monitored along with the ion compositions of soils. The main objective of this study was to understand if the marsh soils containing monotypic stands of native (*Spartina*) and non-native (*Phragmites*) vegetation display similar levels of heavy metals. Differences were observed in the concentrations of heavy metals at study sites with varying marsh vegetation types, and in soils containing vegetation and no vegetation. The soils with dense *Spartina* and *Phragmites* stands were anaerobic whereas soil at the boat ramp site was comparatively less anaerobic and also had increased levels of cadmium. Heavy metal concentrations in soil and *Phragmites* leaves were inversely correlated whereas they were positively correlated in *Spartina* sites. Electrical conductivity and pH levels in soil also showed increased cadmium and arsenic concentrations. These findings collectively infer that human activities and seasonal changes can increase soil complexities affecting the bioavailability of metals.

## Introduction

Mid-Atlantic estuarine wetlands are vital habitats for numerous aquatic organisms including plants, fishes, birds, and mammals. Two hydrophytic plants, the native cordgrass (*Spartina alterniflora*) and the non-native common reed (*Phragmites australis*) predominate these wetlands (1, 2). The aggressive invasion of common reed in the Delaware Bay estuaries has raised concerns on the ecosystem health and the productivity of the affected areas (3–6). It has been reported that anthropogenic activities exacerbate the spread of common reed, and while invasive species are generally considered to have negative impacts on the ecosystems they inhabit. In contrast some studies indicate that the common reed has illustrated the ability to play a key role in ecosystem functions with regards to heavy metal mitigation (6). Reports also indicate that aquatic plants are regularly exposed to pollutants thereby their roots, rhizomes, and other organs could uptake higher concentrations of pollutants and heavy metals (7). This ability of plants, specifically cord grass and the common reed, makes them ideal bio-indicators and focal subjects for pollution mitigation studies (7, 8).

Wetland plants constantly live under inundated conditions increasing the rate of microbial anaerobic respiration (9). This alters the processes of adsorption and desorption of ions in the soil (10) which can affect the bio availability of metals (11). Soils in wetlands are mostly anaerobic and are often reported to have increased concentrations of heavy metals (4). The extent of metal uptake by plants from the soils largely depends on their bioavailability, redox potential, pH and hydrological conditions including the water content (12, 13). Physico-chemical changes in marsh soils can increase the solubility of heavy metals and promote their discharge into aquatic systems and may significantly harm the aquatic life and thus impact the ecology of the system (14). Transport of heavy metals from soil into the aquatic ecosystems therefore depends on the solubility of metals, which is influenced by aerobic or anaerobic conditions, pH, and redox potential (15).

According to United States Environmental Protection Agency (USEPA), mercury, cadmium, lead, nickel, copper, zinc, chromium, and arsenic are the common metal contaminants in soils affected by anthropogenic activities (15, 16). Metal type and their bio availabilities in soils determine the extent of physiological uptake and potential toxic effects of metals in living organisms (17). For example, precipitates and insoluble metal complexes in soils are largely unavailable to plants (18). In brackish wetland ecosystems, the presence of salt ions may reduce the root uptake of metals (11) and impact plant removal efficiency. Overall health of tidal wetlands is heavily reliant on the microorganisms and other organisms that dwell within the ecosystem including crustaceans, fish, and mammals. The concern is that these metal contaminants, even present at low concentrations in the sediments, can bio accumulate in the lower trophic level organisms and could become harmful to consumers at the apex of ecosystem food webs (19). In fact, heavy metal concentrations can reach critical levels in low trophic level organisms such as detritivores. For example, the Atlantic blue crab (*Callinectes sapidus*) is a detritivore that is recreationally and commercially important in the Mid-Atlantic region (20).

Several heavy metals are naturally present in low concentrations in soils and thus could be considered harmless. However, human interferences in natural ecosystems can increase the levels of these metals. Common sources of heavy metals in the study site, Blackbird Creek (BBC) tidal marsh originate from agricultural, residential, transportation and recreational activities (4, 21–23). Metals chosen for this study have known anthropogenic sources: lead (Pb) has residential and recreational sources from drinking water lines, oil, and ammunition, and arsenic (As) from pesticides and fertilizers, and cadmium (Cd) from phosphorous-based fertilizers (24, 25). This is the reason we chose to focus on Arsenic, lead, and mercury in our study. However, these metals have geological (non-anthropogenic) sources as well. This study was conducted to understand how various activities at the study sites can impact ion-heavy metal concentrations and their relations. The focus of this research was to explore if we can find differences in the heavy metal concentrations within the soils of native and non-native vegetation. Results from this research will illustrate environmental significance on how vegetation type can influence the soil quality and ecosystem health.

## Methods

### Study Site

The study site Blackbird Creek (BBC) Estuarine Wetland is located within the Appoquinimink watershed in New Castle County, Delaware. Blackbird Creek is tidally fed from the Delaware Bay to a major extent and flows into the Delaware River. The wetland area has been receiving considerable anthropogenic impacts from residential, agricultural, and recreational activities yet still maintains a relatively pristine classification (26). The site is currently managed and monitored by Delaware National Estuarine Research Reserve (DNERR). This is a unique site that has Major vegetations in the tidal marsh area were identified as cordgrass and common reed.

### Sample Collection

**Figure 1:**
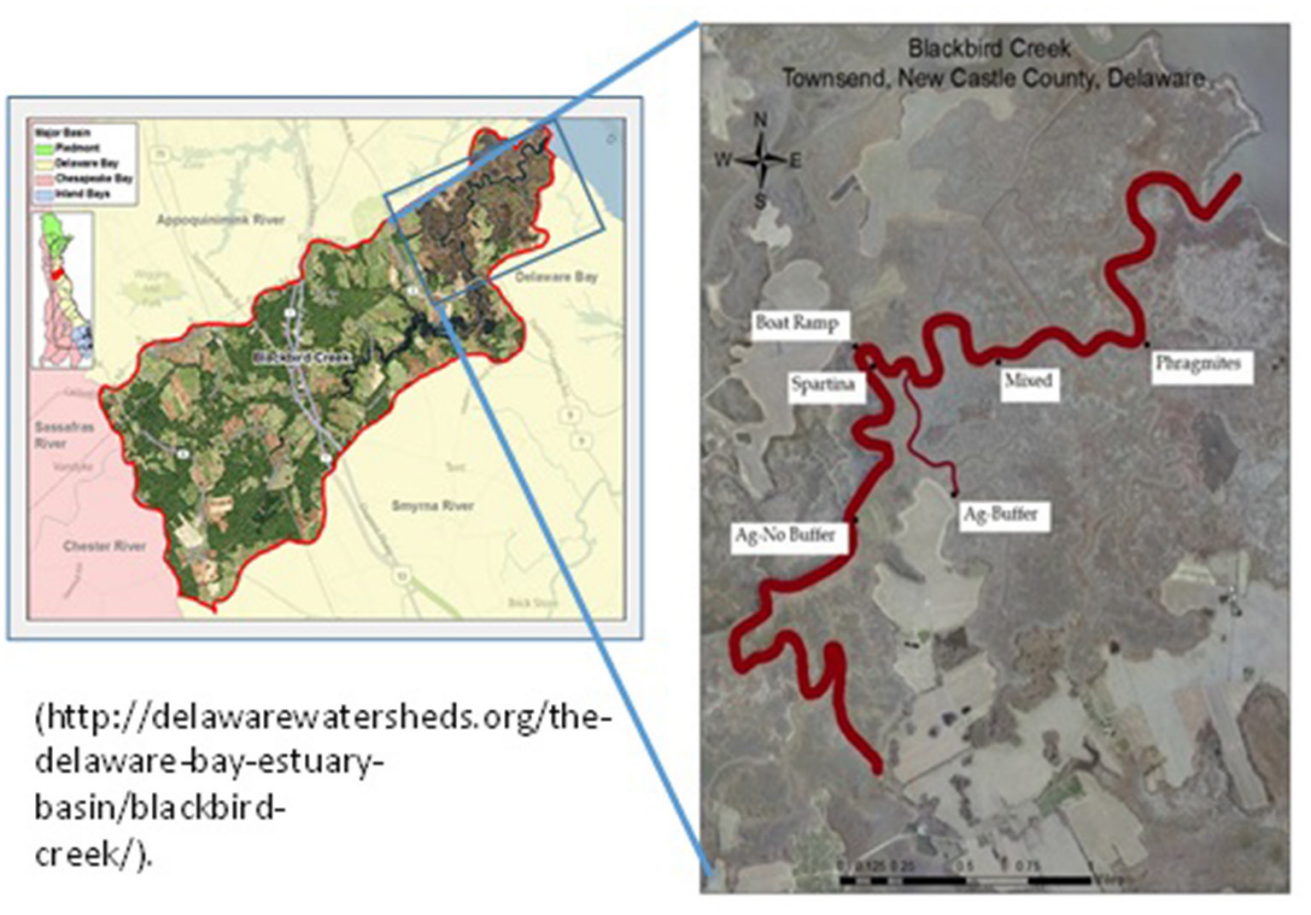
Soil and water sampling sites in the Blackbird Creek, Townsend, Delaware *Phragmites*- (P); mixed site (M); agriculture (Ag-B); boat ramp (BR); *Spartina* (S); agriculture site without buffer (Ag-NB). First map is from DNREC website.

Six sampling sites were randomly selected in the BBC tidal marsh area from the mouth of the creek to the Delaware Bay with varying cordgrass and common reed plant densities: *Phragmites* (P), mixed grass site (M) containing both *Phragmites* and *Spartina*, Agriculture (Ag-B) site with buffer, Boat ramp (BR), *Spartina* (S), and Agriculture site without buffer (Ag-NB) (Figure 1).

#### Soil

The surface plant litter was removed and soil samples from the top 2.5 cm at the six sampling locations were collected monthly from May to November in 2014 and 2015. Soil samples were collected using a clean shovel and placed in labeled one-quart plastic zip-lock bags and kept on ice in a cooler for transportation from the field to the laboratory. Samples were collected monthly and for 2 years to observe the trends in soil nutrients and heavy metal concentrations with relation to human activities. The soil samples were dried at 110°C and grounded to <0.1 mm using a ceramic mortar and pestle.

#### Pore Water

Soil pore water samples were also collected. At each of the six soil sampling sites, a custom-built 30 × 30 cm quadrat was laid next to the soil sampling spots and wet soils were collected from the center and the four corners of the quadrat-outlined area to prepare a composite sample. triplicate samples were collected from each site. The samples in zip-lock bags were stored in a cooler on ice and transported to the laboratory. Pore water samples were collected monthly for 2 years. At the time of analysis aliquots (50 g) of the wet soil sample was transferred into a 50 mL centrifuge tube and centrifuged at 13,000 revolutions per minute (rpm) using a Sorvall high speed centrifuge (Thermofisher Scientific, RC 6+, PA) for 20 minutes to separate pore water from the soil solids according to Guo et al. (27). The isolated pore water was passed through a 0.45-micron nylon filter and analyzed for concentrations of As, Pb, and Cd using inductively coupled plasma-atomic emission spectroscopy (ICP-AES) techniques (IRIS Intrepid II XSP Duo View, Thermo Electron, Franklin, MA).

#### Plants

Common reed and cordgrass leaves were collected from June through September 2014 from several individual plants at each site monthly using clean scissors. The leaves were then placed in labeled plastic bags and stored on ice and transported to the laboratory. After bringing them to the laboratory, the plant samples were frozen in liquid nitrogen and then stored at −80°C to prevent bacterial growth. Leaf samples were cut with scissors into small pieces (20–23 cm) and placed in aluminum foil boats, then dried in the oven at 80°C for 24 h. The dried samples were then ground to <0.1 mm using a motor and pestle. Three grams of the ground sample were weighed in a crucible and then heated at 460°C for 24 h in a Thermo Scientific Thermolyne Muffle Furnace (27). The ashes were cooled to the room temperature, wrapped in Bemis parafilm, and stored in a fume hood until further analysis.

Acid Digestion of the Processed Samples for Heavy Metals Analysis: All tools used for acid digestion were washed with 5% nitric acid, rinsed with deionized water, and air dried.

#### Soils

Soil samples were digested using Parr Microwave Acid Digestion Vessel (PMADV) following the methods of Guo et al. (27). In brief, 1,000 mg of soil sample was weighed into a Polytetrafluoroethylene (PTFE) vial, followed by addition of 3 mL concentrated trace-metal-grade nitric acid and 3 mL High Performance Liquid Chromatography (HPLC)-grade deionized water. The PTFE vial was then loaded into a digestion bomb and heated in a conventional microwave oven (RCA Model, Curtis International Ltd. Etobicoke, Ontario, Canada) at 50% power for 2.5 min. The digestate was fully transferred into a 50 mL volumetric flask.

#### Plant Leaves

Leaf ashes were digested using an alternative acid digestion method (3). Both soil and plant digested samples were filtered through Whatman number two 70 mm filter circles and stored in centrifuge tubes in an acid storage cabinet until analysis.

### Graphite Furnace Atomic Absorption Spectrophotometer (GFAAS) Analysis

The digested soil and leaf samples were analyzed for As, Pb, and Cd concentrations using the Graphite Furnace Atomic Absorption Spectrophotometer (GFAAS) (AAnalyst 600, Perkin Elmer, PA), in three technical triplicates. Winlab 32 software was used for atomization program for each metal analysis. Before analyzing the samples, the instrument was calibrated first using standards and matrix modifiers were used to reduce background noise. For example, palladium was used for As and ammonium phosphate for Cd and Pb. After analysis, a mean concentration from three technical triplicates was calculated for each sample.

### Statistical Analysis

The data was analyzed using statistical software package*, PRIMER 6 (Primer-E Ltd, Plymouth, UK)*. Analysis of similarities (ANOSIM) is an analog of univariate analysis of variance (ANOVA) and is used to analyze the differences in the heavy metal concentrations between the study sites (marsh soil and marsh grasses) and study months. Heavy metal (arsenic, cadmium and lead) data in 2014 for the *Phragmites* and *Spartina* soils and grasses was exported into the PRIMER-E program, these data were normalized, and a resemblance matrix was constructed between the samples using the Euclidean distances. ANOSIM was performed on the resemblance matrix, the factors considered in the analysis were the study sites (*Spartina* soil, *Phragmites* soil, *Spartina* grass and *Phragmites* grass). In this test “R” value varying from 0 to 1, indicates the strength of the factors on the samples. R values close to “0” indicate no separation between the factor groups while R values close to “1” indicate high levels of separation. Principal component analysis (PCA), a multivariate analysis was performed to determine the relationship patterns of heavy metal and ion concentrations during the study period.

## Results and Discussion

### Heavy Metal Concentrations in the Soils

**Figure 2:**
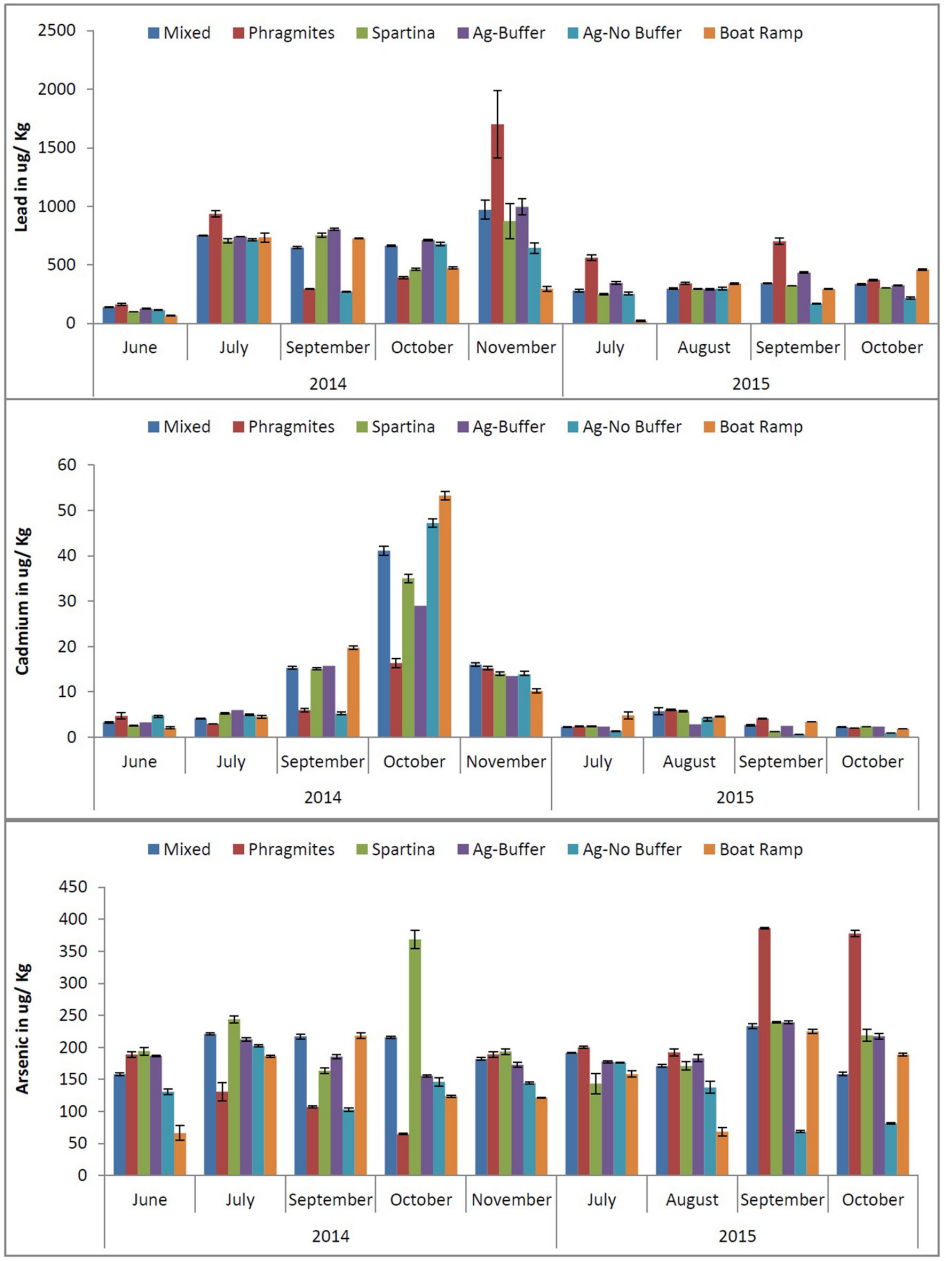
The concentrations of heavy metals (Pb, Cd, and As) in the marsh soils for the six study sites observed during the years 2014 and 2015.

Arsenic concentrations in soils during the two-year study period ranged from 68 to 386 ug/ kg, while lead levels ranged from 67 to 1700 ug/ kg (Figure 2). Cadmium concentrations were comparatively low in the soils of BBC, ranging from 1 to 53 ug/ kg. As illustrated in Figure 2, temporal relationships between two sampling years showed a steady decrease in the concentrations of As, Cd, and Pb at all six study sites. An unusual spike in the Cd concentrations in October of 2014 may be associated with a storm event causing high levels of precipitation and flooding in and around the tidal marsh. There was a spike in Pb concentration in November for *Phragmites* site in 2014 followed by Mixed and Ag Buffer sites. The spike in Pb levels occurred 1 month after Cd spike for Boat Ramp followed by Mixed and Ag-No Buffer sites. This change could be expected as Cd might have been absorbed faster by the plants and the soil while Pb remained relatively intact the soil (28). Cadmium sorption to soil displayed greater pH dependence than Pb, it has been reported that Cd was absorbed via electrostatic surface reactions and/or possible inner-sphere complexation at pH 3.7 (29). In this study, pH at the boat ramp in October was 3.7 which might have resulted in higher and faster Cd absorption. It has been reported that Pb generally adsorbs more strongly than Cd in the soils (29) and poses less of a threat to underlying ground water systems due to its lower mobility and availability. However, the LEAD Group (30) reported that Cd is more readily taken up by plants than other metals such as Pb which can cause Cd concentrations in the soils to reduce.

The soils of monotypic stands of *Phragmites* (common reed) retain the highest levels of Pb than did *Spartina* (cord grass) soils whereas *Spartina* soils had higher levels of Cd than the *Phragmites* soils. Surprisingly, As levels were higher in *Spartina* soils in 2014 compared to *Phragmites*, while As levels of *Phragmites* soils were higher than *Spartina* in 2015. *Spartina* is known to excrete heavy metals through the salt glands present on the surface of its leaves (8). For majority of the study period, the Boat Ramp site had comparatively higher levels of heavy metals than the agricultural sites. More specifically Cd levels were higher in the Boat Ramp soil than all the other study sites. There were no significant trends observed in the levels of heavy metals between the other study sites.

### Heavy Metals in Plant Leaves vs. Soils

**Figure 3:**
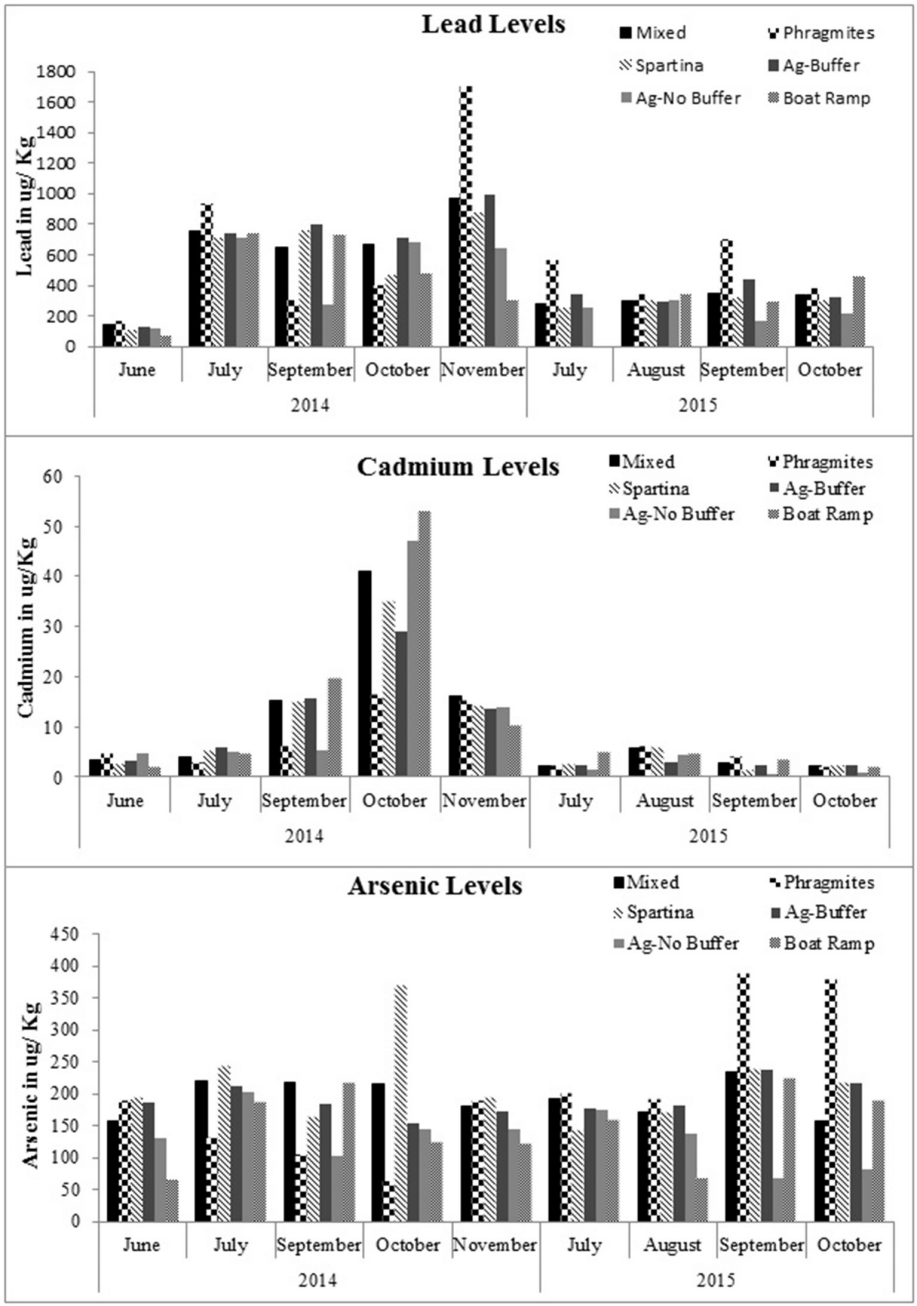
Relationships for lead, cadmium and arsenic concentrations within the marsh soil and *Phragmites* leaves for the study year 2014.

**Figure 4:**
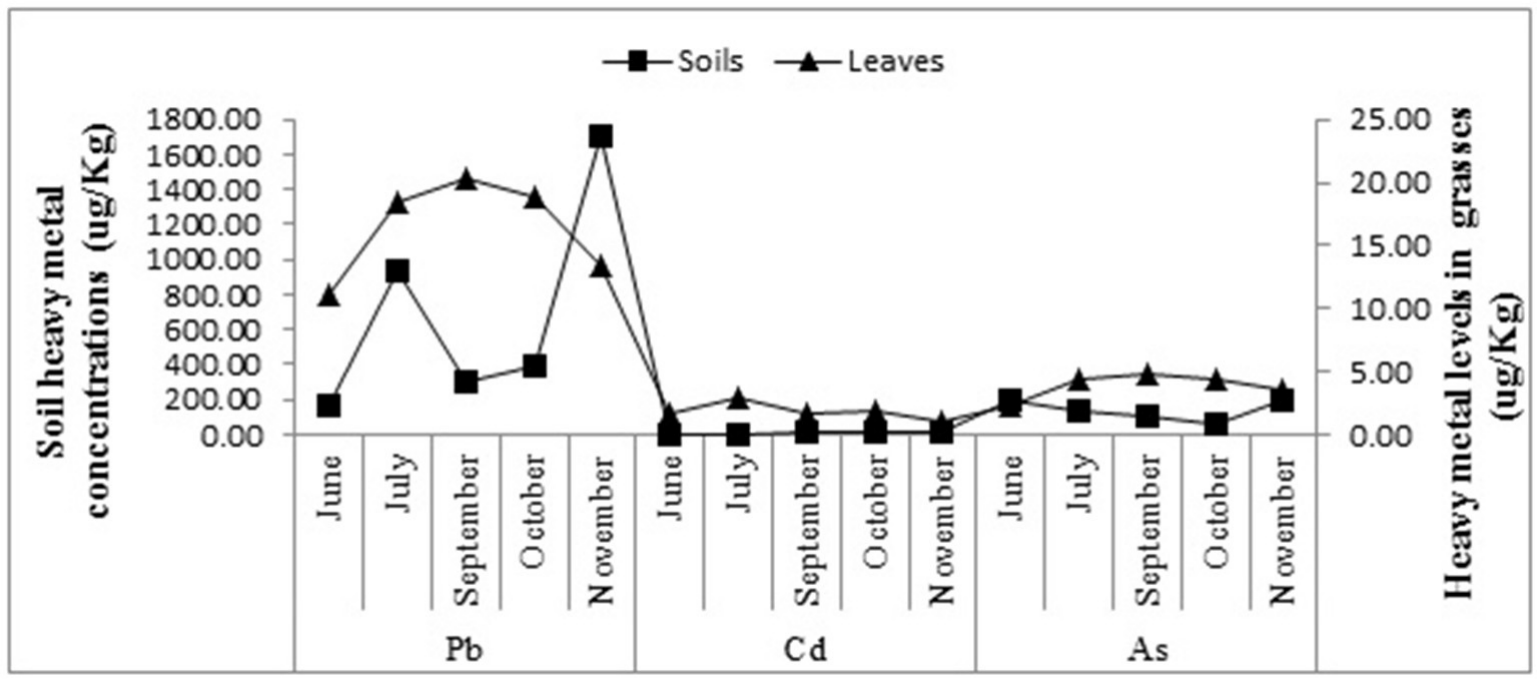
Relationships for lead, cadmium and arsenic concentrations within the marsh soil and *Spartina* leaves for the study year 2014.

Soil samples had much higher heavy metal concentrations than the leaves. Figures 3, 4 illustrate the relationships between As, Pb, and Cd concentrations in the 2014 soil and leaf samples at the *Phragmites* and *Spartina* study sites. At the *Phragmites* site (Figure 3), Pb concentrations in the soils and leaves were compared and there was a parallel increase of Pb in soils and leaves during June (the growing season), following the July samples, the relationship becomes inverse for Cd, As, and Pb. The concentration of Cd and Pb in both soils and leaves had an inverse relationship at the *Spartina* site (Figure 4) from the month of September, while As concentrations seem to have no trends. As shown in Figures 3, 4 during the month of November, the levels of As, Cd, and Pb were higher in soils than in the test plants. Marsh grasses in BBC started to senesce by the end of October or early November, reducing their potential to remove heavy metals from the soils as compared with the growing season. This may be one of the reasons why heavy metal concentrations are high in soils yet less in grasses during November.

ANOSIM results generated a R value equal to 0.389 for the study sites (*Phragmites* and *Spartina*), indicating that the study sites are not much different from each other in regard to the heavy metal concentrations. A P value of 0.001 was generated for this statistical test, suggesting that these results are statistically significant. ANOSIM results for the study months resulted in a R value of −0.073 (which is close to 0), implicating that there are no significant differences in the concentration of heavy metals between the study months, *P* > 0.05; therefore, the results are not statistically different.

Pairwise tests between the study groups (soil vs. grasses) were performed for the sampling time and the R and P values are given in Table 1. These results indicate that there are significant differences in the concentration of heavy metals present at *Spartina* and *Phragmites* grass sites (*R* = 0.64; *P* < 0.05) whereas, there is no significant difference in the heavy metal concentrations within their soils (*R* = −0.02 and *P* > 0.05). But significant differences were observed between *Phragmites* soil vs. *Phragmites* grasses (*R* = 0.53; *P* < 0.05) and *Spartina* soil vs. *Spartina* grasses (R=0.64; P <0.05). There were no significant differences between the study months (*R* = −0.07; *P* = 0.84) for the heavy metals analyzed in the marsh grasses and soils.

**Table 1:** Pairwise comparisons for the heavy metal concentrations between the marsh grasses and marsh soils.

**Groups**	**R value**	**P value**
*Spartina* soil vs. *Phragmites* soil	−0.02	0.516
*Spartina* grass vs. *Phragmites* grass	0.64	0.008
*Phragmites* soil vs. *Phragmites* grass	0.53	0.008
*Spartina* soil *vs. Spartina* grass	0.64	0.008

### Heavy Metals vs. Co-existing Elements in Soils

**Figure 5:**
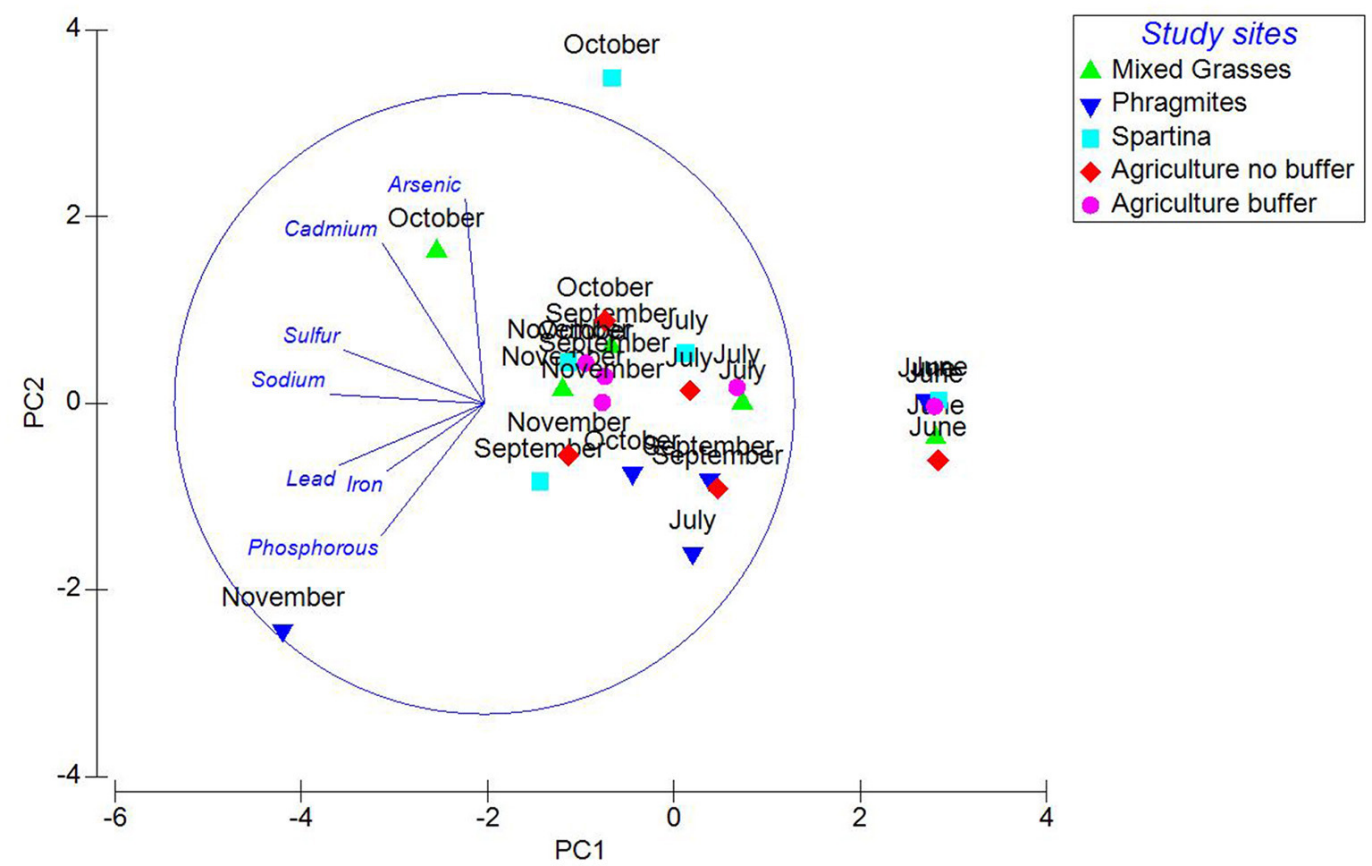
Principal component analysis for heavy metals and ions at study sites with marsh grasses for the year 2014.

Principle Component Analysis (PCA) of soil heavy metals and other co-existing important elements in 2014 displayed a 66% variation among the samples. According to the PCA plot (Figure 5), arsenic, cadmium, sulfur and sodium, in that order had greater effects on the study sites. This plot also showed that when arsenic levels increased, phosphorous levels decreased. Studies report that arsenic competes with phosphorous because both elements in anionic forms are taken by the plant through similar phosphate transporter system (31). The PCA plot also displays that there are no differences between the variables tested for the study months and the sites. But soil samples from the *Spartina* and mixed sites in October had higher levels of arsenic while the mixed site also had higher levels of cadmium. In November, some soil samples from the *Phragmites* site had high levels of phosphorous, while all variables were high during June at all study sites. Generally, *Phragmites* and *Spartina* start dying in October, thus the plants do not use phosphorous for their growth which thereby increases phosphorous in the soils. Phosphorous levels were low in the *Spartina* site in comparison to the other sites (Table 2). Also, there was little difference in the soil phosphorous level between the agricultural sites with and without a buffer zone. June samples are clustered separately; this might be because this month is considered as early growth season where fertilizers might have been sprayed. In October 2014, sampling for the soil samples was performed following a hurricane event and this might have impacted the levels of metals and the co-existing salt components at the study sites. This PCA plot also explains that as the iron and phosphorous levels decrease in the soils, the lead levels decrease accordingly.

**Table 2:** The concentrations of heavy metals and the ion compositions for the pore water samples in 2014.

**Samples**	**Lead**	**Cadmium**	**Arsenic**	**Sulfur**	**Calcium**	**Sodium**	**Phosphorous**	**Iron**	**pH**	**EC**
	**ug/kg**	**ug/kg**	**ug/kg**	**(mg/l)**	**(mg/l)**	**(mg/l)**	**(mg/l)**	**(mg/l)**		**(mmhos/cm)**
Mixed –J	752.5	4.08	220.83	166.99	84.4	1,170	0.04062	0.06296	6.4	7.75
Mixed –S	649.63	15.29	217	363	163.69	2,596	0.0634	0.07925	4	15.46
Mixed-O	664.4	41.08	215.67	742	263.36	2,066	0.06824	0.29897	4.1	14.05
Mixed-N	971.24	16	181.9	329.8	152.99	3,256	0.04514	0.05356	6.3	18.1
Phragmites-J	935.83	2.94	130.66	146.65	75.9	1,103	0.08	0.24	4.2	7.75
Phragmites-S	296.7	5.96	106.97	305.8	144.81	2,477	0.02	0.11	4.2	15.11
Phragmites-O	393.03	16.35	64.43	561.4	204.39	2,208	0.06	0.06	6.1	14.35
Phragmites-N	1,702.57	15.22	189.13	402.4	162.05	3,132	0.67	0.22	3.1	18.31
Spartina-J	708.6	5.31	243.83	366.7	152.5	1,335	0.01	0.12	5.1	8.93
Spartina-S	753.2	15.08	163.43	262.5	143.64	2,291	0.01	0.61	3.9	14.24
Spartina-O	460.97	35.01	368.33	316	143.05	2,408	0	0.04	5.2	14.9
Spartina-N	874.1	14.01	193.57	522.5	179.79	2,518	0.02	0.08	4	15.78
Ag No-Buffer-J	716.4	4.92	202.1	419.8	145.7	1,254	0.01554	0.07459	4.7	8.57
Ag No-Buffer-S	272.97	5.2	102.75	307.5	150.56	2,366	0.02934	0.10734	6.5	14.33
Ag No-Buffer-O	679.27	47.16	146.03	195.51	125.8	1,690	0.06122	0.11737	7.2	10.8
Ag No-Buffer-N	645.73	13.89	144.1	394.2	159.9	2,565	0.02195	0.35267	3.9	15.8
Ag Buffer-J	742.33	5.96	212.5	222.6	97.49	1,177	0.00402	0.05717	5.3	8.18
Ag Buffer-S	803.07	15.7	185.5	389.8	152.9	2,412	0.03898	0.07163	4	14.64
Ag Buffer-O	712.1	28.94	155.27	326.6	146.36	2,514	0.03629	0.11748	4.2	14.81
Ag Buffer-N	996.92	13.5	172.7	381.6	140.6	2,339	0.03268	0.03673	6.1	14.23
Boat Ramp-J	735.67	4.53	186.43	192.25	136.4	1,748	0.073	0.1	7.3	11.5
Boat Ramp-S	726.14	19.69	218.13	266.4	137.05	2,605	0.04	0.11	6.7	15.27
Boat Ramp-O	475.2	53.23	123.67	519.5	215.48	3,530	0.42	0.82	3.7	19.55
Boat Ramp-N	295.83	10.18	121.57	ISS	ISS	ISS	ISS	ISS	ISS	ISS

*Results are provided for the months we sampled. J, July; S, September; O, October; N, November; Ag, Agriculture; ISS, Insufficient sample*.

The pore water pH, electrical conductivity (EC), salt components (sulfur, calcium, iron, sodium, phosphorous) and heavy metals are presented in Table 2. The pH values ranged from 3.1 to 7.3; the spatial variations observed among the study sites may be due to their pH and ion levels. The pH of samples decreased in September but increased in October at all study sites except for boat ramp and agriculture site without buffer. These sites contained less vegetation compared to the other study sites. These results are in consistence with previous studies (32) indicating that more oxidizing reactions occur in areas with vegetation thereby decreasing the pH. The protons generated by the oxidation reactions neutralize alkalinity of the water surrounding soil solid particles and consequently, lowered the pH (33, 34). Per our results, pH of soils might have been increased in October because the samples in this month were collected after the hurricane Gonzalo, which might have caused the soils to flood with storm water causing in pH changes. This pH increase can be observed more prominently in the sites with *Phragmites* (4.2–6.1) which is closer to the mouth of the bay.

**Figure 6:**
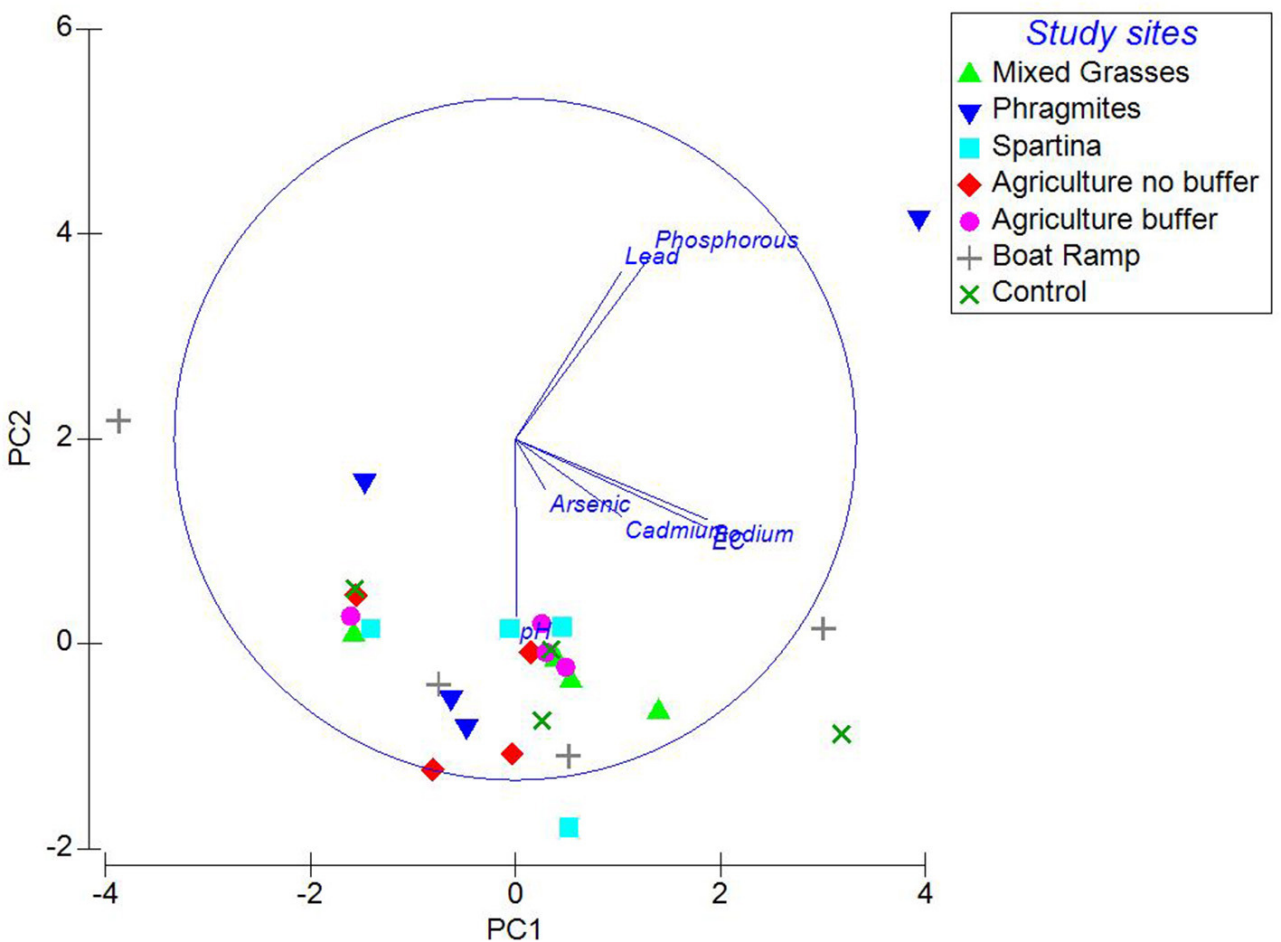
Relationship between electrical conductivity, pH and phosphorous and the heavy metal concentrations of the soil samples.

PCA analysis shows that the variables such as sodium, EC, and pH are closely associated with arsenic and cadmium while lead and phosphorous were closely associated with each other (Figure 6). This indicates that when phosphorous levels increased in soils, lead levels increased and when sodium, EC levels increased in the soils then arsenic and cadmium levels increased. The pH values were comparatively lower in the *Spartina* sites than the other study sites. This might explain that the bioavailability of metals in soils to these marsh grasses is greatly altered because of pH, EC, and co-existing salt ions. It has been reported that acidity of soils has a greater impact on the bioavailability of heavy metals (35).

As shown in Figure 6, EC and salinity were directly proportional to the levels of arsenic and cadmium in soil samples. Our study results agree with previous studies by McLaughlin et al., Lin et al., Muhlingh et al. (36–38), in which cadmium levels were increased in potatoes, sunflower and wheat under increased saline conditions, even though soil cadmium levels were low. It has been mentioned that an elevated salinity enhances the solubility of heavy metals, as salt-derived anions react with heavy metals and thereby, increase the competition between the salt-derived cations and heavy metals for their adsorption to soil particles (39, 40). As shown in Figure 6, and the EC (salinity) of soils is high which means there are more soluble Na^+^ and Cl^−^ ions in the soil that can readily react with cadmium forming soluble complexes such as cadmium chloride (41).

**Figure 7:**
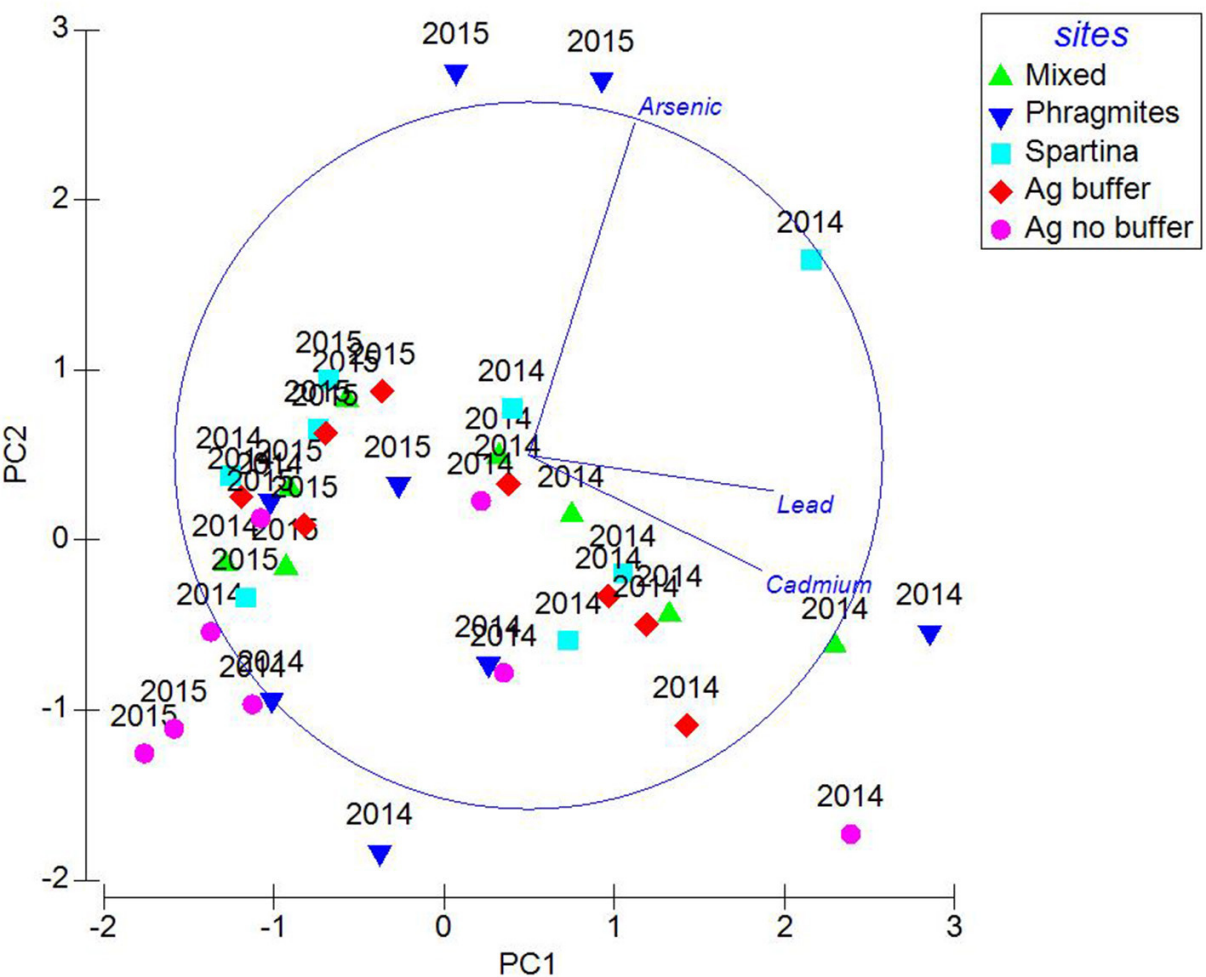
Principal component analysis of heavy metals at the marsh grass sites for the years 2014 and 2015.

**Figure 8:**
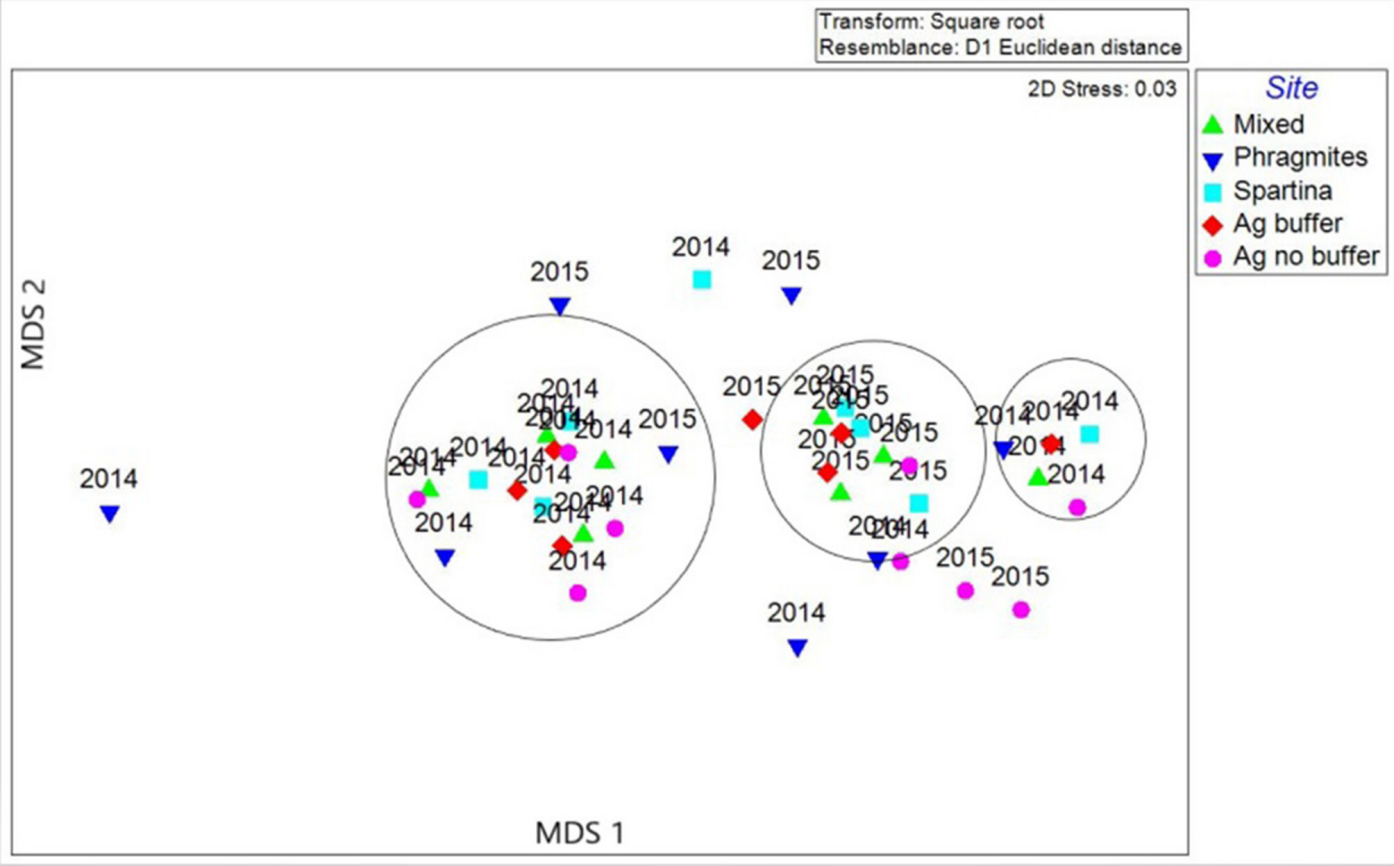
Multidimensional analysis to study the similarities of the study sites for heavy metals during 2014 and 2015.

Heavy metal concentrations were higher in year 2014 than 2015 (Figure 7). A resemblance matrix of the heavy metal data for 2014 and 2015 has been generated and MDS plots were created based on the Euclidean distances to study the relationships of the study sites in both study years. The MDS plot (Figure 8) shows that even though the data points from 2014 and 2015 are close, groupings were observed among the samples. This shows that the heavy metal concentrations in the samples from 2014 were different from those in 2015. The MDS plot with study site analysis shows that the data points from all the study sites are in close proximity in relation to the year (2014 and 2015). But the data points from the *Phragmites* site are more scattered than those of the *Spartina* site, which infers that a higher degree of dissimilarity exists between them. MDS plots in relation to months show that the samples from June 2014 have formed as a separate group and are distant from other 2014 samples. This confirms that the heavy metal concentrations in June are different from those in the other months. The results from MDS analysis are in consistence with the PCA analysis.

The stress values generated for this plot is 0.03, indicating an excellent fit for the data points. The amount of stress generated from the MDS plot interprets the quality of analysis and whether the analysis is suitable for the input data. Any stress values <0.025 is considered as an excellent fit (42, 43). Salt marsh estuaries are complex ecosystems. Studies show that the roots of marsh grasses carry diverse bacteria that can breakdown the humic acids and other compounds in the soil under changing pH and other characteristics, thereby altering the mobility and solubility of metal complexes (44, 45).

Our study results also show that the levels of sodium and sulfur were greater than iron and phosphorous at the study sites. It can be interpreted from the results that arsenic and phosphorous share inverse relationships. Studies suggest when arsenic uptake increases in plants, increased levels of phosphorous can be observed in the soil as both arsenic and phosphorous share similar phosphate transporter systems (46, 47). The solubility of most heavy metals is highly pH dependent (48). High alkaline pH and low electrical conductivity reduce the solubility of certain metals like zinc, cadmium, and copper because they may be precipitated as hydroxides or carbonates (49–52).

## Conclusion

The present study results reveal both direct and inverse relationships between the heavy metal compositions in the soils and marsh plant leaves. The inverse relationships found at the *Phragmites* site seem to follow the growing seasonal patterns.

In conclusion, the type of metal up taken by the plants or insoluble metal complexes formed in the soil are all governed by the nature of the study site, soil characteristics, type of the vegetation at the site, weather conditions and human activities occurring within the ecosystem. Also, microorganisms that harbor in the roots of marsh grasses change depending on the type of plant species and this may impact the oxidation-reduction potential of soil nutrients. In addition, the season of the year can impact the availability of the heavy metals for the plants or their abundance in the soil because temperature, salinity and pH greatly shift their distribution and concentrations according to the season. Fertilizers used during the cropping season can alter the nutrient levels in the soil as they compete with heavy metal complexes making them unavailable to the plant such as relationship between phosphorus and arsenic. Thus, complex interactions occur in the soil specifically in tidal marshes where the environment continuously changes. In our study, relationships of ions to heavy metal concentrations explain complex relationships that are being supported by other researchers. Future studies will focus on the detailed analysis of pore water ions and heavy metals in relation to molecular assessment to understand the connection between the ion transport mechanisms to the levels of heavy metals in plants and soils.

## Data Availability Statement

The datasets presented in this study can be found in online repositories. The names of the repository/repositories and accession number(s) can be found in the article/supplementary material.

## Author Contributions

LC planned this research, designed the experiment, trained the second author (undergraduate student), conducted the experiment, analyzed the results, and wrote the manuscript. KO collected the soil and plant samples, conducted the experiments, and was involved in writing the manuscript. GO was involved with initial field testing, planning, sample collection, student and staff training and supervision, laboratory logistics for analysis and obtaining resources for the project, and preparing the manuscript. All authors contributed to the article and approved the submitted version.

## Conflict of Interest

The authors declare that the research was conducted in the absence of any commercial or financial relationships that could be construed as a potential conflict of interest.

## Publisher's Note

All claims expressed in this article are solely those of the authors and do not necessarily represent those of their affiliated organizations, or those of the publisher, the editors and the reviewers. Any product that may be evaluated in this article, or claim that may be made by its manufacturer, is not guaranteed or endorsed by the publisher.
